# Solid and cystic intrapancreatic accessory spleen: report of 10 cases in a single institution

**DOI:** 10.1080/07853890.2025.2463564

**Published:** 2025-02-10

**Authors:** Jianjie Sheng, Yifei Yang, Yudong Qiu, Chenglin Lu, Xu Fu

**Affiliations:** ^a^ Department of Pancreatic and Metabolic Surgery, Nanjing Drum Tower Hospital Clinical College of Nanjing University of Chinese Medicine, Nanjing, China; ^b^ Department of Pancreatic and Metabolic Surgery, Nanjing Drum Tower Hospital, Affiliated Hospital of Medical School, Nanjing University, Nanjing, China

**Keywords:** Accessory spleen, pancreatic mass, ECIPAS, case series

## Abstract

**Background:**

Precise diagnosis of intrapancreatic accessory spleen (IPAS) remains challenging due to its rarity and diverse presentations. Despite comprehensive examinations, including radiography and other diagnostic methods, the potential for malignancy cannot be excluded, often leading to unnecessary pancreatic surgeries. We review our institutional experience to provide insights for accurately distinguishing IPAS.

**Methods:**

We retrospectively reviewed 10 patients who underwent distal pancreatectomy for the lesion in the pancreas tail which was determined to be IPAS on final pathology at our institution between January 2020 and April 2024. The presenting symptoms, medical history, preoperative imaging, operative therapy, final pathology and postoperative course were evaluated.

**Results:**

Patient ages ranged from 30 to 72 (median 55.5), including six women and four men. Most patients were asymptomatic. One patient had the medical history of splenectomy. Lesions ranged from 1.4 to 7.3 cm (mean 2.9 cm). All lesions were located in the pancreatic tail. On radiologic evaluation, these lesions had both solid and cystic presentations. The most common operative approach was laparoscopic distal pancreatectomy and splenectomy. Four patients were diagnosed with epidermoid cysts arising in intrapancreatic accessory spleen (ECIPAS) on final pathologic evaluation.

**Conclusions:**

IPAS are predominantly benign lesions which have solid and cystic presentations commonly mistaken for pancreatic neoplasms. Combining CT, MRI, EUS-FNA and nuclear medicine may enhance IPAS detection, though no definitive diagnostic method exists. Increased awareness of IPAS in the differential diagnosis of pancreatic tail tumors, coupled with advancements in imaging techniques could improve diagnostic accuracy and exclude malignancy, preventing unnecessary surgeries.

## 1. Introduction

An accessory spleen (AS), or splenunculus, is a lobule of splenic tissue observed in ectopic locations. The majority of ASs are located in the splenic hilum (62.1%). Other locations are less common but may be misdiagnosed as tumours, especially when localized in or near the pancreas (5.5%) [1]. Splenic tissue is highly vascularized, and radiographically, its hypervascularity often makes it difficult to distinguish from non-functioning pancreatic neuroendocrine tumours (NF-PNET) or hyper­vascular metastatic tumours [2]. Sometimes, the cystic-appearing presentation of IPAS can be mistaken for pancreatic cystic neoplasms [3]. Given the diverse presentation of IPAS, though sufficient examinations including radiography and other diagnostic methods have been completed, malignant potential cannot be excluded. In most cases, patients would suffer unnecessary pancreatic surgeries. Unfortunately, available literature cannot provide solid recommendations regarding preoperative differential diagnosis of IPAS and pancreatic tumours.

In this study, we retrospectively reviewed our experience with IPAS in 10 patients who underwent pancreatic resection. By examining the clinical presentation, radiologic imaging, preoperative diagnosis, treatment and final pathologic results of these patients, and integrating insights from the existing literature, we attempt to provide some clues for accurately diagnosing IPAS.

## 2. Methods

We retrospectively reviewed patients who underwent distal pancreatectomy with or without splenectomy for a pancreatic mass suspected to be malignant or indeterminate between pancreatic and splenic diseases, and upon final pathologic evaluation, were diagnosed with intrapancreatic accessory spleen (IPAS) at our institution between 2020 and 2024. The presenting symptoms, medical history, preoperative imaging, operative therapy, final pathology and postoperative course were evaluated. Clinical data were collected from the hospital record system. Pathological data were collected from the Department of Pathology. All patients were informed about the study and provided consent to participate. Written informed consent for publication of their data was obtained from all participants and the study was approved by the Health Research Ethics Board of Drum Tower Hospital of Nanjing University Medical School (2024-794-01). The participants agreed to the use of their data and its publication in the article.

## 3. Results

**Figure 1.**
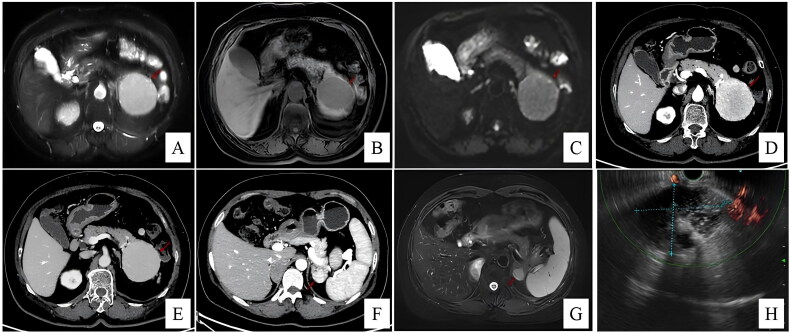
Patient 8: in T2-weighted image (A), the cystic lesion (arrow) is hyperintense in the pancreatic tail. The mass is clearly seen as a hypointense lesion (arrow) in the T1-weighted image (B). Diffusion-weighted MR imaging shows a restriction (C). In arterial (D) and portal-venous phase (E), the lesion displays hyperintense (arrow). Patient 7: slight heterogeneous enhancement which is similar to the spleen in the arterial phase (F); the signal for the lesion (arrow) is similar to that of the spleen in the T2-weighted image (G). Patient 10: a hypovascular cystic lesion between the pancreas tail and the spleen, with no enhanced cystic wall nodules and no ductal dilation (H).

A total of 10 patients were included in the study. Their clinical, radiologic, operative and post-operative features are demonstrated in Table 1. Patient ages ranged from 30 to 72 with a median age of 55.5, including six women and four men. One patient had the medical history of splenectomy (case 8, Figure 1). Three patients presented with symptoms initially, while the others were asymptomatic. Lesions ranged from 1.4 to 7.3 cm in maximal diameter with a mean of 2.9 cm. All lesions were located in the pancreatic tail.

**Table 1.** Patient demographics and clinical characteristics (*N* = 10).

Demographics	
Age (years)	55.5 (30.0–72.0)
Gender (female, %)	6 (60%)
Clinical history	
Presenting symptoms, *n* (%)	
Asymptomatic	7 (70%)
Abdominal pain/bloating	3 (30%)
Hypertension, *n* (%)	4 (40%)
Diabetes mellitus, *n* (%)	3 (30%)
Coronary artery disease, *n* (%)	1 (10%)
Hepatitis B, *n* (%)	2 (20%)
Previous surgical history, *n* (%)	
Appendectomy	2 (20%)
Splenectomy	1 (10%)
None	7 (70%)
Abnormal tumour markers, *n* (%)	3 (30%)
Preoperative imaging findings	
Tumour size (cm)	2.9 (1.4–7.3)
Tumour location (pancreatic tail), *n* (%)	10 (100%)
Character of the mass, *n* (%)	
Solid	5 (50%)
Cystic	3 (30%)
Mixed	2 (20%)
Preoperative suspicion of IPAS, *n* (%)	
CT/MRI suspected AS	1 (10%)
US suspected AS	1 (10%)
EUS suspected ECIPAS	1 (10%)
Preoperative diagnosis, *n* (%)	
PNET	6 (60%)
MCN	2 (20%)
SPT	1 (10%)
SCN	1 (10%)
Surgical approach, *n* (%)	
Laparoscopic	6 (60%)
Pathologic diagnosis, *n* (%)	
AS	6 (60%)
ECIPAS^a^	4 (40%)
Postoperative complications, *n* (%)	4 (40%)

^a^ The immunohistochemical results showed: case 5: GATA3(+++), TTF-1(−), P63(+++), P40(+++), CK19(+++), MUC2(−), MUC6(−), MUC5ac (−) and CDX-2(+). Case 10: CK7(+), P63(+), Ki67(2%), CK5/6(+), D2-40(−), CD31(−), CR (−), CD4(−), CD163(+) and CD8(+).

**Figure 2.**
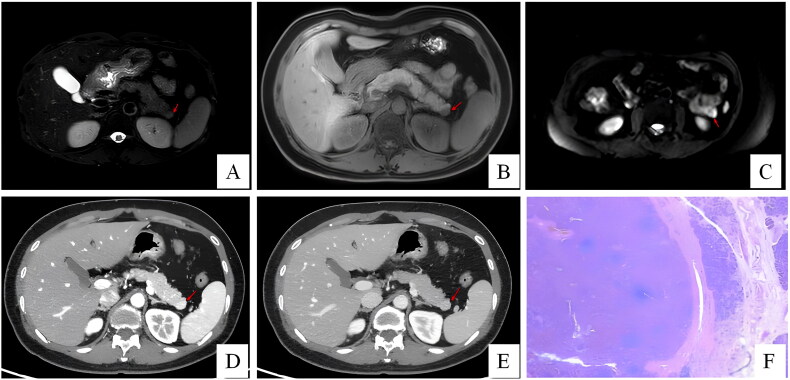
Patient 4 was diagnosed with NET preoperative. In T2-weighted image (A), the mass is hardly visible as a slightly hyperintense lesion in the pancreatic tail (arrow). Hypointense lesion (arrow) in T1-weighted image (B). The lesion (arrow) presents high signal intensity in the DWI image, and septations are faintly visible (C). In arterial (D) and portal-venous phase (E), the tumour is hardly visible displaying slightly hyperintense (arrow). Histological (F) images of intrapancreatic accessory spleen. Blood sinus structures, and lymphoid follicles and sinusoid-like structures can be seen.

On radiologic evaluation, these lesions had solid and cystic presentations. Lesions usually display well-circumscribed, hypervascular or cystic masses in the tail of the pancreas. Solid lesions were typically identified as round, hypervascular, well-circumscribed masses on CT and MRI, with enhancement patterns and signals similar to those of the spleen (Figure 2). Cystic lesions were usually well‑defined, hypovascular and contained septations, without enhanced cystic wall nodules or ductal dilation.

**Figure 3.**
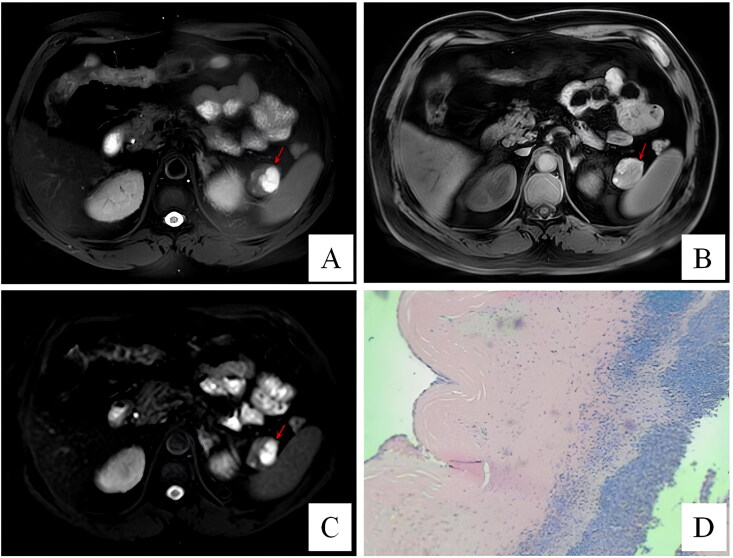
Patient 3 was diagnosed with SCN preoperative. In T2-weighted image (A), a hyperintense lesion with enhanced septations (arrow) is seen in the pancreatic tail. The lesion (arrow) is still hyperintense in the T1-weighted image (B). In the diffusion-weighted image(C), the lesion (arrow) has high signal intensity. Histological (D) images of epidermoid cyst in intrapancreatic accessory spleen (ECIPAS). Cysts were covered by stratified squamous epithelium.

**Figure 4.**
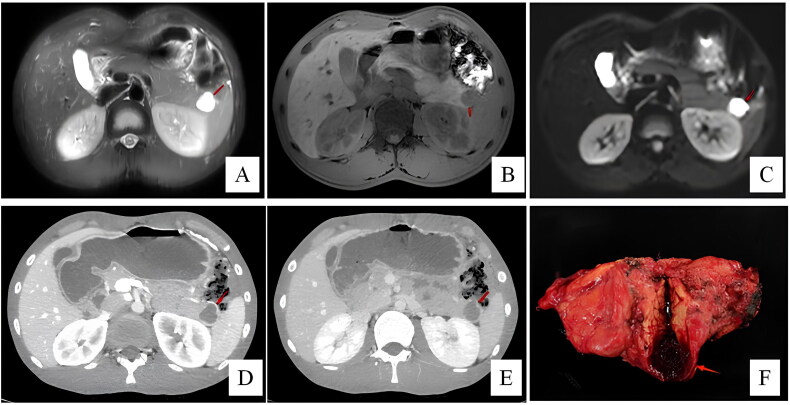
Patient 5 was diagnosed with MCN preoperative. In T2-weighted image (A), a well-demarcated cystic lesion (arrow) is seen in the pancreatic tail. The lesion (arrow) is hypointense in the T1-weighted image (B). The lesion (arrow) presents high signal intensity in DWI image (C). In arterial (D) and portal-venous phase (E), the lesion is hypointense (arrow). Macroscopic (F) image of epidermoid cyst in intrapancreatic accessory spleen (ECIPAS).

**Figure 5.**
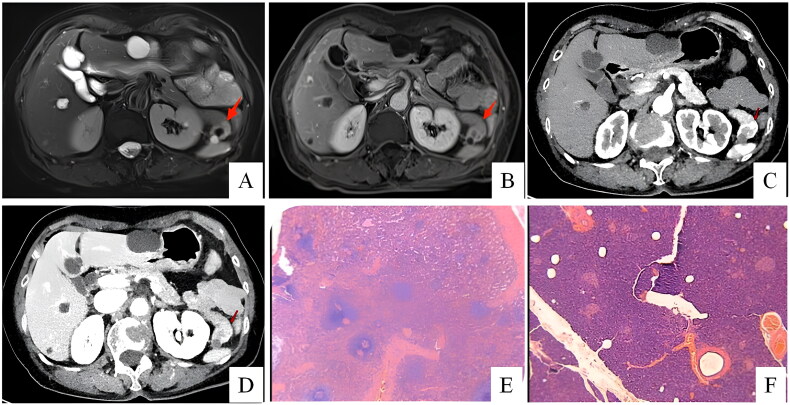
Patient 9 was diagnosed with SPT preoperative. In T2-weighted image (A), a well-demarcated cystic lesion (arrow) is seen in the pancreatic tail. The lesion (arrow) displays lobulated and hypointense in the T1-weighted image (B). In arterial (C) and portal-venous phase (D), the tumour displays as a well-circumscribed cystic lesion with intra-calcification. The solid portion is well-enhanced (arrow). Histological (E, F) images of ECIPAS.

Six patients were suspicious of NET before surgery. Four patients were diagnosed with pancreatic cystic neoplasms, including one SCN (case 3, Figure 3), two MCN (case 5, Figure 4; case 10) and one SPT (case 9, Figure 5). One patient (case 5) underwent EUS-FNA before surgery, which returned negative for malignant cells. Preoperative radiologic examinations suggested AS in case 7 (CT, MRI), case 8 (US) and case 10 (EUS), but malignancy could not be definitively excluded (Figure 1).

Six patients underwent laparoscopic surgery, with the most common operative approach being laparoscopic distal pancreatectomy (either with or without splenectomy). The average intraoperative blood loss was 120 ml. Four patients experienced postoperative complications in the postoperative hospital course. Three patients (cases 1, 5 and 8) developed CD II organ/space SSI, and one patient (case 6) developed a urinary tract infection. The median length of postoperative hospital stay was 8.5 days (range 6–12 days).

Four patients (cases 3, 5, 9 and 10) were diagnosed with ECIPAS on final pathologic evaluation. The immunohistochemical (IHC) results for case 5 were as follows: GATA3(+++), TTF-1(−), P63(+++), P40(+++), CK19(+++), MUC2(−), MUC6(−), MUC5ac (−) and CDX-2(+). The IHC results for case 10 were: CK7(+), P63(+), Ki67(2%), CK5/6(+), D2-40(−), CD31(−), CR (−), CD4(−), CD163(+) and CD8(+).

## 4. Discussion

In this study, we present a case series of 10 patients who underwent pancreatic resection but were ultimately diagnosed with IPAS upon final pathologic evaluation, including both solid and cystic forms. Despite relatively sufficient preoperative examinations for pancreatic tumours had been done, some indicating the possibility of an AS, a definitive diagnosis of IPAS and the exclusion of potential malignancy still could not be established, leading to the decision for surgical intervention. The accurate diagnosis of IPAS remains challenging due to its changeable presentation. Given its rarity and diverse manifestations, there is no consensus on its epidemiologic features, pathogenesis, clinical manifestations, tumour biomarker abnormalities or imaging characteristics.

IPAS are mostly benign tissue masses, occurring in 10–15% of the general population [4]. The appearance of IPAS differs, and only a small fraction can be recognized radiographically. IPAS’s hypervascularity often makes them indistinguishable from NF-PNETs or hypervascular metastatic tumours. Besides, IPAS occasionally have cystic-appearing presentations including epidermoid cysts arising in intrapancreatic accessory spleen (ECIPAS), cavernous hemangioma in the intrapancreatic accessory spleen (CHIPAS), e.g. as have been reported [3,5]. Cystic-appearing IPAS are rare. In the general population, the prevalence of ECIPAS is 1.7% [3]. These cystic formations can be easily mistaken for pancreatic cystic neoplasms. Therefore, IPAS should be included in the differential diagnosis of pancreatic tail tumours, especially for those who have a splenectomy medical history (case 8). And the possibility of originating from B-cell lymphoma should also be taken into consideration [6].

Accessory spleens arise from the incomplete fusion of splenic tissue located in the dorsal mesogastrium during the fifth week of embryologic development [7]. Multiple mechanisms may contribute to the formation of ECIPAS. Sumida et al. proposed that the origin of ECIPAS might differ from that of pancreatic ductal origin, as indicated by the detection of Pbx1 expression in epithelial cells and the presence of lipid accumulation in the intra-cystic fluid through immunohistochemistry [8]. However, the pathogenesis of ECIPAS, including the origin of its cystic epithelium and its relationship with splenic epidermoid cyst (SEC), is still not well established. Exploring the pathological features of IPAS could offer valuable insights into its origins.

Most patients in our study exhibited normal levels of biomarkers typically used to monitor pancreatic tumours. The low phase-positive predictive value of biomarkers currently used to monitor pancreatic tumours including CA19-9, CEA, CA125 and the low specificity of neuroendocrine markers indicate that serological markers are not effective in the differential diagnosis between IPAS and NET or pancreatic carcinoma [9].

When it comes to the differential diagnosis of pancreatic masses, CT is often the first choice. IPAS’s solitary, well-defined, hypervascular presentation on contrast-enhanced computed tomography (CE-CT) is consistent with the radiological characteristics of pNET [2,10]. Despite the similar attenuation observed on both non-contrast and post-contrast scans, and the heterogeneous enhancement pattern during the arterial phase resembling that of the spleen, which helps identify IPAS on CE-CT (case 7) [9], its detection remains challenging. Additional MRI is needed to help to clarify the diagnosis. On MRI, solid IPAS typically exhibits high signal intensity (SI) on T2-WI and low SI on T1-WI. Certain pancreatic tumours, particularly PNET, share imaging characteristics with IPAS [11]. Different from pancreatic tumours, IPAS has a low apparent diffusion coefficient (ADC) value, high DWI SI and inhomogeneous enhancement on the arterial phase similar to the spleen (case 7) [9,11]. However, the fact that IPAS is rarely considered in differential diagnosis by radiologists and surgeons complicates its identification. Additionally, not all IPAS have the same SI with the spleen. Diffusion-weighted MRI (DWI) and SPIO-enhanced MRI can add important clues to conventional CE-MRI. Both absolute and normalized ADC values can facilitate the differentiation between IPAS and PNET [12]. CT and MRI used in combination with DWI are important in the diagnosis of IPAS, especially for solid IPAS and the solid portion of the cystic kind.

On the abdominal US, IPAS has its specific presence of the vascular hilum into which the vessels enter. Compared with the US, EUS has higher resolution and sensitivity. On EUS, IPAS typically appears as a round, homogenous lesion with clear and regular boundaries. It usually displays a homogeneous, green elastographic pattern on EUS-elastography [13]. The similar profiles of PNETs on EUS make it still difficult to make a diagnosis excluding PNET with this imaging modality. Though the bridge of spleen tissue connecting the lesion to the spleen which could be observed by dynamic real-time EUS imaging may help distinguish IPAS from PNETs [14], diagnosing IPAS by EUS criteria alone remains inaccurate. Biopsy via EUS or ESD is often needed to improve the accuracy of the diagnosis [13,15]. Immunohistochemical techniques are increasingly utilized to distinguish between IPAS and PNET. Among a variety of markers used in the pathological differential diagnosis, CD8 is more valuable [16]. Therefore, CD8 staining should be included in PNET-related immunohistochemistry. Among three patients whose preoperative radiologic examinations suggested IPAS, one ECIPAS patient (case 10) had CEH-EUS findings that demonstrated a hypovascular cystic lesion between the pancreas tail and the spleen, with no enhanced cystic wall nodules and no ductal dilation, indicating the suspicious diagnosis of ECIPAS. Minami et al. reported a case of ECIPAS diagnosed preoperatively by CE-EUS and EUS-FNA, thus avoiding surgical resection [17]. These findings suggest that EUS and EUS-FNA may be useful for diagnosing cystic IPAS (e.g. ECIPAS). On the other hand, for minor pancreatic lesions, especially those smaller than 5 mm, where only the surrounding epidermoid cystic fluid can be aspirated, FNA has no advantage [18,19]. Moreover, the sensitivity and specificity of FNA are limited due to the variable accuracy of imaging guidance and the non-specific cytological features of IPAS.

The awareness to include IPAS in the differential diagnosis of pancreatic tail tumours is important. Correlative imaging is linked with clinical suspicion [11]. When a well-circumscribed solid lesion in the distal pancreas appears similar to the spleen on CT or MRI, or when a well-defined cystic lesion is located at the edge of the pancreatic tail or between the pancreatic tail and spleen – without enhanced cystic wall nodules or ductal dilation – clinicians should especially consider the possibility of solid or cystic IPAS, particularly in patients with a history of splenectomy, and proceed with further evaluation. Multimodality imaging is essential in the diagnosis of IPAS.

PET/CT is proven to enhance the accuracy of diagnosis. 68-Ga PET/CT plays a crucial role in differentiating PNETs from IPASs. Its capability to differentiate PNET mainly depends on the high expression of somatostatin receptor (SSTR, mainly SSTR2 and SSTR5) [2]. Different radiotracers target specific SSTR subtypes, resulting in varying diagnostic efficacy. However, instances of false-positive results in Ga-68 PET/CT have been reported, where IPASs are misdiagnosed as pNETs [20,21]. Due to the physiologic high spleen uptake of [^68^Ga] Ga-DOTATATE [22], Liberini et al. have introduced a novel technique for correcting ^68^Ga-Dotatate PET/CT data and more effectively distinguishing PNETs from IPASs [23]. Furthermore, dynamic ^68^Ga-DOTATATE PET-CT shows promise as a potential tool for the evaluation of pNETs [24].

For the prominent physiological uptake of up to 90% of injected Tc-99m heat-denatured red blood cells (HDRBCs) in functional splenic tissue, this imaging modality has been demonstrated to be a superior choice for differentiating PNETs from IPASs [25]. However, false negatives also may occur when IPAS is smaller than 2 cm or when the spleen has minimal function [10]. Therefore, to diagnose and localize an AS more precisely, Tc-99m HDRBC single photon emission computed tomography (SPECT) is better combined with other cross-sectional imaging modalities [9]. But when it comes to the cystic type (e.g. ECIPAS), where the solid portion is compressed by cysts and the proportion of functional splenic tissue is minimal [17], the diagnostic value of this technique is still limited. Its time-consuming nature and the need for direct handling of blood products also limit this technique’s widespread use across most hospitals [11].

**Figure 6.**
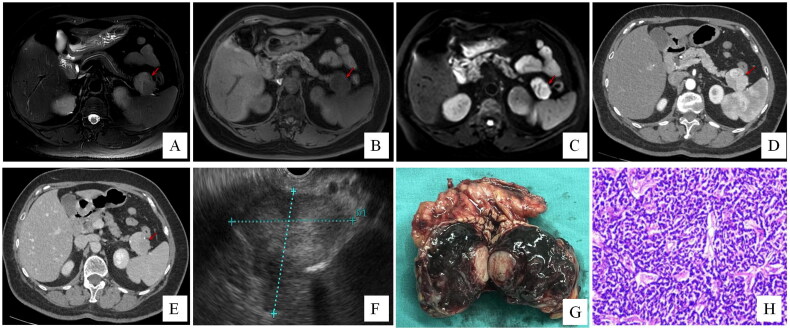
A patient who was considered PNET but the possibility of IPAS could not be excluded preoperatively. The final patho­logy confirmed a PNET. In T2-weighted image (A), a cystic-solid lesion (arrow) in the pancreatic tail. The mass is a hypointense lesion (arrow) in the T1-weighted image (B). Diffusion-weighted MR imaging presents high signal intensity (C). In arterial (D) and portal-venous phase (E), the lesion demonstrates marked heterogeneous enhancement (arrow). EUS revealed a lesion in the splenic hilum, with a differential diagnosis of an AS or a NET (F); macroscopic (G) image of dissected surgical specimen. Histological (H) image suggests a grade 2 PNET.

Nevertheless, the variability of pancreatic tumours and IPAS continues to pose challenges for definitive differential diagnosis. Just as demonstrated by a recent case of NET with a rare presentation at our centre, where IPAS was considered in the preoperative differential diagnosis (Figure 6). Increasing awareness of IPAS, coupled with continuous advancements in imaging modalities, can improve the likelihood of detecting IPAS. But there remains no definitive diagnostic method. Therefore, the need for the development of novel imaging techniques or new applications of existing diagnostic methods to enhance the accuracy of IPAS diagnosis is pressing. A new imaging analysis technique, radiomics (or texture analysis), shows potential for distinguishing PNETs from IPASs [26]. Recently, Ahmed and Fishman implied a new way to help differential diagnosis – cinematic rendering. This 3D postprocessing technology provides superb surface detail and textural perception [27]. And in some conditions, the size of an IPAS may imitate changes in the main spleen’s size. This phenomenon can sometimes indicate the presence of an IPAS. The main spleen and AS share the same circulation, hormonal influences and functional roles. Consequently, pathological changes in the main spleen are often replicated in the AS. For example, IPAS may enlarge in patients with ITP who undergo splenectomy [28]. Similarly, in haemolytic conditions which cause spleen enlargement, the size of the IPAS might decrease following the reduction of the main spleen size after medical treatment. In contrast, the sizes of other pancreatic tumours do not decrease with immunosuppressive treatment. Therefore, these changes in IPAS size can sometimes support its diagnosis [29].

Obtaining the correct diagnosis of IPAS preoperatively through non-invasive means is important. The continuous evolution and advancement of imaging techniques, particularly in the field of nuclear medicine, are largely driven by a deeper understanding of the pathogenesis and histopathology of IPAS. The imaging of IPAS is related to its various histologic compositions [9]. This kind of histologic–radiologic correlation may provide valuable insights for developing new techniques. Further understanding of histological features will also improve the diagnostic accuracy of established methods, such as FNA biopsy and IHC analysis. Exploring the pathogenesis of IPAS, with a focus on its histopathological features, may contribute to the advancement of novel non-invasive imaging modalities. The development of a robust animal model and genetic profiling will be crucial in testing and validating current hypotheses regarding its pathogenesis [8].

IPAS is innocuous in nature and does not require treatment [12]. Only when IPAS combined with recurrent hypersplenism or ITP, malignancy or potential malignancy, causing compression or with torsion or rupture, surgery is needed [15]. Additionally, if littoral cell angioma (LCA) is detected in the main spleen, both the main spleen and the AS should be removed, regardless of the AS’s pathological characteristics [30]. In clinical practice, given the malignancy and poor prognosis of pancreatic neoplasms, it is reasonable to perform surgical resection to ensure long-term survival if the possibility of a pancreatic tumour fails to be definitively ruled out.

## 5. Conclusions

IPAS are mostly benign lesions which have solid and cystic presentations commonly mistaken for pancreatic neoplasms. Solid IPAS tend to be well-circumscribed, hypervascular and share the same presentation on CT or MRI with the spleen. Cystic IPAS are generally well‑defined, hypovascular, with septations, with no enhanced cystic wall nodules and no ductal dilation. Combining CT, MRI, EUS-FNA and nuclear medicine examinations may improve the likelihood of detecting IPAS. However, a definitive diagnostic method for IPAS remains elusive. The exploration of novel imaging techniques or new applications of existing diagnostic methods is needed. Increasing awareness of including IPAS in the differential diagnosis of pancreatic tail tumours, alongside ongoing advancements in imaging technologies, holds promise for enhancing the definitive diagnosis of IPAS and completely excluding any potential malignancy, thereby preventing unnecessary surgeries without any lingering concerns.

## Data Availability

The data and materials are not publicly available due to patient privacy concerns, but are available from the corresponding author upon reasonable request.
